# Supplementary material for: Multi‐dataset identification of innovative feature genes and molecular mechanisms in keratoconus
Source: J Cell Mol Med. 2024 Sep 19;28(18):e70079. doi: 10.1111/jcmm.70079 (PMC11412914; doi:10.1111/jcmm.70079)
Supplement: Supplementary file 2 — Material S2. [file JCMM-28-e70079-s002.zip › 7.GSEA analysis/2.ATOH7/KEGG_PATHWAYS_IN_CANCER.html]

Details for gene set KEGG\_PATHWAYS\_IN\_CANCER[GSEA]

|  || Dataset | ATOH7.group.cls#h\_versus\_l |
| Phenotype | group.cls#h\_versus\_l |
| Upregulated in class | h |
| GeneSet | KEGG\_PATHWAYS\_IN\_CANCER |
| Enrichment Score (ES) | 0.3341551 |
| Normalized Enrichment Score (NES) | 1.413235 |
| Nominal p-value | 0.053742804 |
| FDR q-value | 0.28754136 |
| FWER p-Value | 0.892 |
Table: GSEA Results Summary

  

Fig 1: Enrichment plot: KEGG\_PATHWAYS\_IN\_CANCER      
 Profile of the Running ES Score & Positions of GeneSet Members on the Rank Ordered List

  

| SYMBOL | TITLE | RANK IN GENE LIST | RANK METRIC SCORE | RUNNING ES | CORE ENRICHMENT || 1 | AKT3 | na | 9 | 1.043 | 0.0250 | Yes |
| 2 | WNT1 | na | 172 | 0.697 | 0.0372 | Yes |
| 3 | MITF | na | 236 | 0.652 | 0.0512 | Yes |
| 4 | AXIN2 | na | 285 | 0.630 | 0.0651 | Yes |
| 5 | NKX3-1 | na | 301 | 0.624 | 0.0798 | Yes |
| 6 | NOS2 | na | 363 | 0.597 | 0.0925 | Yes |
| 7 | VEGFC | na | 373 | 0.591 | 0.1065 | Yes |
| 8 | MMP1 | na | 439 | 0.567 | 0.1184 | Yes |
| 9 | WNT9A | na | 551 | 0.534 | 0.1281 | Yes |
| 10 | FGFR1 | na | 573 | 0.527 | 0.1402 | Yes |
| 11 | LEF1 | na | 735 | 0.492 | 0.1475 | Yes |
| 12 | MMP9 | na | 811 | 0.476 | 0.1568 | Yes |
| 13 | WNT6 | na | 825 | 0.474 | 0.1680 | Yes |
| 14 | RXRG | na | 913 | 0.462 | 0.1766 | Yes |
| 15 | SHH | na | 937 | 0.457 | 0.1870 | Yes |
| 16 | WNT5B | na | 1090 | 0.433 | 0.1931 | Yes |
| 17 | BCL2 | na | 1098 | 0.432 | 0.2034 | Yes |
| 18 | ETS1 | na | 1232 | 0.416 | 0.2096 | Yes |
| 19 | EGLN3 | na | 1322 | 0.406 | 0.2169 | Yes |
| 20 | CSF2RA | na | 1707 | 0.368 | 0.2146 | Yes |
| 21 | COL4A6 | na | 2104 | 0.337 | 0.2113 | Yes |
| 22 | PRKCB | na | 2281 | 0.324 | 0.2140 | Yes |
| 23 | PPARG | na | 2303 | 0.323 | 0.2212 | Yes |
| 24 | PGF | na | 2372 | 0.317 | 0.2269 | Yes |
| 25 | CCNA1 | na | 2422 | 0.314 | 0.2331 | Yes |
| 26 | SMO | na | 2428 | 0.314 | 0.2406 | Yes |
| 27 | FZD7 | na | 2482 | 0.310 | 0.2466 | Yes |
| 28 | FGF4 | na | 2628 | 0.302 | 0.2497 | Yes |
| 29 | WNT9B | na | 2657 | 0.300 | 0.2561 | Yes |
| 30 | LAMA1 | na | 2673 | 0.300 | 0.2630 | Yes |
| 31 | BMP4 | na | 2853 | 0.288 | 0.2648 | Yes |
| 32 | ZBTB16 | na | 2887 | 0.286 | 0.2707 | Yes |
| 33 | TRAF1 | na | 2930 | 0.284 | 0.2764 | Yes |
| 34 | WNT11 | na | 3016 | 0.280 | 0.2807 | Yes |
| 35 | TGFB1 | na | 3047 | 0.278 | 0.2866 | Yes |
| 36 | TCF7 | na | 3059 | 0.277 | 0.2930 | Yes |
| 37 | FZD10 | na | 3060 | 0.277 | 0.2997 | Yes |
| 38 | BMP2 | na | 3243 | 0.268 | 0.3009 | Yes |
| 39 | WNT7A | na | 3390 | 0.261 | 0.3030 | Yes |
| 40 | WNT8B | na | 3424 | 0.259 | 0.3083 | Yes |
| 41 | STAT5A | na | 3484 | 0.257 | 0.3128 | Yes |
| 42 | PDGFB | na | 3642 | 0.250 | 0.3143 | Yes |
| 43 | RAD51 | na | 3872 | 0.241 | 0.3135 | Yes |
| 44 | GLI1 | na | 4085 | 0.233 | 0.3130 | Yes |
| 45 | MAP2K1 | na | 4240 | 0.228 | 0.3140 | Yes |
| 46 | FGF23 | na | 4389 | 0.222 | 0.3151 | Yes |
| 47 | FZD2 | na | 4571 | 0.216 | 0.3151 | Yes |
| 48 | CXCL8 | na | 4857 | 0.206 | 0.3118 | Yes |
| 49 | CDKN1A | na | 5247 | 0.193 | 0.3052 | Yes |
| 50 | LAMB3 | na | 5412 | 0.188 | 0.3049 | Yes |
| 51 | APC | na | 5634 | 0.182 | 0.3029 | Yes |
| 52 | COL4A4 | na | 5676 | 0.180 | 0.3061 | Yes |
| 53 | CRKL | na | 5867 | 0.175 | 0.3048 | Yes |
| 54 | WNT7B | na | 5905 | 0.174 | 0.3079 | Yes |
| 55 | GLI2 | na | 5995 | 0.171 | 0.3095 | Yes |
| 56 | RUNX1 | na | 6118 | 0.168 | 0.3100 | Yes |
| 57 | PIK3R3 | na | 6166 | 0.166 | 0.3127 | Yes |
| 58 | MET | na | 6191 | 0.165 | 0.3160 | Yes |
| 59 | RELA | na | 6248 | 0.164 | 0.3183 | Yes |
| 60 | FLT3LG | na | 6249 | 0.164 | 0.3223 | Yes |
| 61 | WNT10A | na | 6547 | 0.156 | 0.3174 | Yes |
| 62 | NFKB2 | na | 6590 | 0.155 | 0.3200 | Yes |
| 63 | TCF7L1 | na | 6644 | 0.153 | 0.3221 | Yes |
| 64 | ITGA6 | na | 6683 | 0.152 | 0.3247 | Yes |
| 65 | FGF13 | na | 6783 | 0.149 | 0.3254 | Yes |
| 66 | FGF6 | na | 6809 | 0.148 | 0.3283 | Yes |
| 67 | SLC2A1 | na | 6979 | 0.144 | 0.3268 | Yes |
| 68 | FAS | na | 7283 | 0.136 | 0.3213 | Yes |
| 69 | RUNX1T1 | na | 7399 | 0.133 | 0.3212 | Yes |
| 70 | WNT3A | na | 7555 | 0.129 | 0.3199 | Yes |
| 71 | CDK6 | na | 7568 | 0.129 | 0.3226 | Yes |
| 72 | LAMA3 | na | 7593 | 0.128 | 0.3250 | Yes |
| 73 | BCL2L1 | na | 7603 | 0.128 | 0.3279 | Yes |
| 74 | NRAS | na | 7693 | 0.126 | 0.3284 | Yes |
| 75 | PRKCA | na | 7938 | 0.120 | 0.3242 | Yes |
| 76 | FZD4 | na | 8076 | 0.117 | 0.3230 | Yes |
| 77 | ITGA2 | na | 8085 | 0.117 | 0.3256 | Yes |
| 78 | WNT2 | na | 8209 | 0.114 | 0.3248 | Yes |
| 79 | CTNNA3 | na | 8333 | 0.112 | 0.3240 | Yes |
| 80 | CUL2 | na | 8424 | 0.110 | 0.3240 | Yes |
| 81 | LAMC1 | na | 8509 | 0.108 | 0.3242 | Yes |
| 82 | TGFB3 | na | 8550 | 0.107 | 0.3256 | Yes |
| 83 | TRAF5 | na | 8600 | 0.106 | 0.3268 | Yes |
| 84 | VEGFA | na | 8615 | 0.106 | 0.3289 | Yes |
| 85 | KITLG | na | 8664 | 0.105 | 0.3301 | Yes |
| 86 | NFKB1 | na | 8680 | 0.105 | 0.3322 | Yes |
| 87 | DCC | na | 8701 | 0.104 | 0.3342 | Yes |
| 88 | FGFR2 | na | 8886 | 0.101 | 0.3313 | No |
| 89 | ITGAV | na | 8959 | 0.100 | 0.3316 | No |
| 90 | CRK | na | 8997 | 0.099 | 0.3329 | No |
| 91 | DVL2 | na | 9335 | 0.093 | 0.3254 | No |
| 92 | LAMC2 | na | 9360 | 0.093 | 0.3269 | No |
| 93 | HGF | na | 9373 | 0.092 | 0.3288 | No |
| 94 | EGLN1 | na | 9417 | 0.092 | 0.3298 | No |
| 95 | ITGB1 | na | 9539 | 0.090 | 0.3284 | No |
| 96 | FGF1 | na | 9677 | 0.087 | 0.3266 | No |
| 97 | TGFA | na | 9931 | 0.083 | 0.3212 | No |
| 98 | CDK4 | na | 9992 | 0.082 | 0.3214 | No |
| 99 | EPAS1 | na | 10024 | 0.081 | 0.3225 | No |
| 100 | CSF1R | na | 10042 | 0.081 | 0.3240 | No |
| 101 | EP300 | na | 10138 | 0.079 | 0.3231 | No |
| 102 | ARAF | na | 10150 | 0.079 | 0.3247 | No |
| 103 | TPR | na | 10221 | 0.078 | 0.3246 | No |
| 104 | HSP90B1 | na | 10223 | 0.078 | 0.3264 | No |
| 105 | CSF3R | na | 10326 | 0.076 | 0.3253 | No |
| 106 | GRB2 | na | 10441 | 0.075 | 0.3238 | No |
| 107 | NFKBIA | na | 10503 | 0.073 | 0.3238 | No |
| 108 | ARNT2 | na | 10666 | 0.071 | 0.3208 | No |
| 109 | APC2 | na | 10749 | 0.070 | 0.3201 | No |
| 110 | ITGA3 | na | 10756 | 0.070 | 0.3216 | No |
| 111 | WNT4 | na | 10868 | 0.068 | 0.3201 | No |
| 112 | PTK2 | na | 10929 | 0.067 | 0.3199 | No |
| 113 | EGLN2 | na | 11504 | 0.059 | 0.3047 | No |
| 114 | MLH1 | na | 11553 | 0.058 | 0.3047 | No |
| 115 | IL6 | na | 11566 | 0.058 | 0.3058 | No |
| 116 | STAT3 | na | 11584 | 0.058 | 0.3067 | No |
| 117 | HIF1A | na | 11625 | 0.057 | 0.3069 | No |
| 118 | TGFBR1 | na | 11693 | 0.057 | 0.3063 | No |
| 119 | CKS1B | na | 11730 | 0.056 | 0.3066 | No |
| 120 | MSH6 | na | 11757 | 0.056 | 0.3072 | No |
| 121 | KIT | na | 11952 | 0.053 | 0.3029 | No |
| 122 | FZD8 | na | 11987 | 0.053 | 0.3032 | No |
| 123 | FGF21 | na | 12140 | 0.051 | 0.3000 | No |
| 124 | COL4A2 | na | 12318 | 0.048 | 0.2960 | No |
| 125 | LAMB1 | na | 12334 | 0.048 | 0.2967 | No |
| 126 | RXRB | na | 12340 | 0.048 | 0.2978 | No |
| 127 | EGFR | na | 12485 | 0.046 | 0.2947 | No |
| 128 | PIAS2 | na | 12594 | 0.045 | 0.2926 | No |
| 129 | CDKN1B | na | 12681 | 0.044 | 0.2912 | No |
| 130 | PIAS3 | na | 12700 | 0.044 | 0.2917 | No |
| 131 | FGF14 | na | 12830 | 0.042 | 0.2890 | No |
| 132 | DVL3 | na | 12971 | 0.041 | 0.2859 | No |
| 133 | HDAC2 | na | 13042 | 0.040 | 0.2849 | No |
| 134 | FGF2 | na | 13152 | 0.039 | 0.2826 | No |
| 135 | JAK1 | na | 13201 | 0.038 | 0.2822 | No |
| 136 | RARA | na | 13254 | 0.038 | 0.2816 | No |
| 137 | DVL1 | na | 13325 | 0.037 | 0.2804 | No |
| 138 | CDKN2A | na | 13514 | 0.035 | 0.2758 | No |
| 139 | TRAF6 | na | 13633 | 0.034 | 0.2732 | No |
| 140 | EGF | na | 13899 | 0.031 | 0.2663 | No |
| 141 | RAC2 | na | 13916 | 0.031 | 0.2666 | No |
| 142 | BIRC5 | na | 14003 | 0.031 | 0.2648 | No |
| 143 | TFG | na | 14049 | 0.030 | 0.2642 | No |
| 144 | CASP9 | na | 14196 | 0.029 | 0.2607 | No |
| 145 | HSP90AB1 | na | 14217 | 0.028 | 0.2608 | No |
| 146 | BIRC3 | na | 14296 | 0.028 | 0.2592 | No |
| 147 | GLI3 | na | 14449 | 0.026 | 0.2554 | No |
| 148 | MSH2 | na | 14476 | 0.026 | 0.2553 | No |
| 149 | MAPK1 | na | 14536 | 0.025 | 0.2542 | No |
| 150 | HRAS | na | 14559 | 0.025 | 0.2542 | No |
| 151 | PLD1 | na | 14651 | 0.024 | 0.2521 | No |
| 152 | TP53 | na | 14698 | 0.024 | 0.2513 | No |
| 153 | FGF12 | na | 14791 | 0.023 | 0.2492 | No |
| 154 | WNT8A | na | 15047 | 0.021 | 0.2423 | No |
| 155 | RAF1 | na | 15219 | 0.019 | 0.2378 | No |
| 156 | PML | na | 15223 | 0.019 | 0.2382 | No |
| 157 | STK36 | na | 15369 | 0.018 | 0.2344 | No |
| 158 | RHOA | na | 15382 | 0.018 | 0.2345 | No |
| 159 | LAMB2 | na | 15383 | 0.018 | 0.2349 | No |
| 160 | IKBKG | na | 15619 | 0.016 | 0.2285 | No |
| 161 | AXIN1 | na | 15654 | 0.015 | 0.2279 | No |
| 162 | JUP | na | 15727 | 0.015 | 0.2261 | No |
| 163 | AKT1 | na | 15787 | 0.014 | 0.2248 | No |
| 164 | GSK3B | na | 16028 | 0.013 | 0.2181 | No |
| 165 | FGF10 | na | 16172 | 0.011 | 0.2142 | No |
| 166 | PIK3CD | na | 16202 | 0.011 | 0.2136 | No |
| 167 | CYCS | na | 16260 | 0.011 | 0.2122 | No |
| 168 | RAC1 | na | 16331 | 0.010 | 0.2105 | No |
| 169 | BRCA2 | na | 16341 | 0.010 | 0.2104 | No |
| 170 | FGF19 | na | 16438 | 0.009 | 0.2079 | No |
| 171 | ABL1 | na | 16492 | 0.009 | 0.2065 | No |
| 172 | ITGA2B | na | 16498 | 0.009 | 0.2066 | No |
| 173 | LAMC3 | na | 16551 | 0.008 | 0.2053 | No |
| 174 | FH | na | 16561 | 0.008 | 0.2052 | No |
| 175 | STK4 | na | 16565 | 0.008 | 0.2054 | No |
| 176 | JUN | na | 16587 | 0.008 | 0.2049 | No |
| 177 | FZD6 | na | 16592 | 0.008 | 0.2050 | No |
| 178 | VEGFB | na | 16644 | 0.008 | 0.2037 | No |
| 179 | GSTP1 | na | 16750 | 0.007 | 0.2008 | No |
| 180 | RALB | na | 16792 | 0.006 | 0.1998 | No |
| 181 | MAPK3 | na | 16821 | 0.006 | 0.1991 | No |
| 182 | FGF11 | na | 16966 | 0.005 | 0.1951 | No |
| 183 | PPARD | na | 17078 | 0.004 | 0.1919 | No |
| 184 | MAPK8 | na | 17140 | 0.003 | 0.1902 | No |
| 185 | RALA | na | 17168 | 0.003 | 0.1895 | No |
| 186 | BAD | na | 17218 | 0.002 | 0.1881 | No |
| 187 | FGF16 | na | 17343 | 0.001 | 0.1846 | No |
| 188 | BAX | na | 17508 | 0.000 | 0.1798 | No |
| 189 | FGF22 | na | 17511 | 0.000 | 0.1798 | No |
| 190 | E2F3 | na | 17541 | -0.000 | 0.1789 | No |
| 191 | SUFU | na | 17833 | -0.003 | 0.1705 | No |
| 192 | DAPK2 | na | 17853 | -0.003 | 0.1700 | No |
| 193 | CDC42 | na | 17874 | -0.003 | 0.1695 | No |
| 194 | FGF20 | na | 17878 | -0.003 | 0.1695 | No |
| 195 | RASSF1 | na | 17883 | -0.003 | 0.1695 | No |
| 196 | SMAD3 | na | 18063 | -0.005 | 0.1644 | No |
| 197 | CDK2 | na | 18103 | -0.005 | 0.1634 | No |
| 198 | HHIP | na | 18105 | -0.005 | 0.1634 | No |
| 199 | NCOA4 | na | 18201 | -0.006 | 0.1608 | No |
| 200 | CTNNB1 | na | 18214 | -0.006 | 0.1606 | No |
| 201 | STAT5B | na | 18496 | -0.008 | 0.1527 | No |
| 202 | E2F1 | na | 18613 | -0.010 | 0.1495 | No |
| 203 | BCR | na | 18703 | -0.010 | 0.1472 | No |
| 204 | DAPK3 | na | 18798 | -0.011 | 0.1447 | No |
| 205 | CTNNA1 | na | 18820 | -0.011 | 0.1444 | No |
| 206 | HDAC1 | na | 19008 | -0.013 | 0.1393 | No |
| 207 | FGF7 | na | 19070 | -0.014 | 0.1378 | No |
| 208 | CCDC6 | na | 19434 | -0.017 | 0.1277 | No |
| 209 | MAX | na | 19465 | -0.017 | 0.1272 | No |
| 210 | TRAF2 | na | 19649 | -0.018 | 0.1223 | No |
| 211 | MYC | na | 19651 | -0.019 | 0.1228 | No |
| 212 | CDH1 | na | 19700 | -0.019 | 0.1218 | No |
| 213 | FOXO1 | na | 19979 | -0.022 | 0.1143 | No |
| 214 | FZD9 | na | 19998 | -0.022 | 0.1143 | No |
| 215 | CBLC | na | 20019 | -0.022 | 0.1142 | No |
| 216 | MSH3 | na | 20028 | -0.022 | 0.1145 | No |
| 217 | WNT3 | na | 20041 | -0.022 | 0.1147 | No |
| 218 | RALBP1 | na | 20136 | -0.023 | 0.1125 | No |
| 219 | TGFBR2 | na | 20164 | -0.023 | 0.1123 | No |
| 220 | CHUK | na | 20245 | -0.024 | 0.1106 | No |
| 221 | FZD3 | na | 20400 | -0.026 | 0.1067 | No |
| 222 | BID | na | 20508 | -0.027 | 0.1043 | No |
| 223 | CCND1 | na | 20546 | -0.027 | 0.1039 | No |
| 224 | MAP2K2 | na | 20653 | -0.028 | 0.1015 | No |
| 225 | PIK3CB | na | 20685 | -0.028 | 0.1012 | No |
| 226 | PIAS1 | na | 20777 | -0.029 | 0.0993 | No |
| 227 | PIAS4 | na | 20832 | -0.030 | 0.0985 | No |
| 228 | MAPK10 | na | 20989 | -0.032 | 0.0947 | No |
| 229 | RASSF5 | na | 21102 | -0.033 | 0.0922 | No |
| 230 | BIRC2 | na | 21238 | -0.034 | 0.0891 | No |
| 231 | FZD5 | na | 21276 | -0.035 | 0.0889 | No |
| 232 | TPM3 | na | 21305 | -0.035 | 0.0889 | No |
| 233 | HSP90AA1 | na | 21322 | -0.035 | 0.0893 | No |
| 234 | PIK3CA | na | 21359 | -0.036 | 0.0891 | No |
| 235 | APPL1 | na | 21460 | -0.037 | 0.0871 | No |
| 236 | RBX1 | na | 21760 | -0.040 | 0.0794 | No |
| 237 | RXRA | na | 21798 | -0.041 | 0.0793 | No |
| 238 | FADD | na | 21903 | -0.042 | 0.0773 | No |
| 239 | SMAD4 | na | 22118 | -0.044 | 0.0722 | No |
| 240 | TCF7L2 | na | 22134 | -0.044 | 0.0728 | No |
| 241 | PIK3R2 | na | 22209 | -0.045 | 0.0718 | No |
| 242 | PDGFA | na | 22257 | -0.046 | 0.0715 | No |
| 243 | FZD1 | na | 22280 | -0.046 | 0.0720 | No |
| 244 | SOS2 | na | 22550 | -0.049 | 0.0653 | No |
| 245 | RB1 | na | 22702 | -0.051 | 0.0622 | No |
| 246 | XIAP | na | 22713 | -0.051 | 0.0631 | No |
| 247 | CTBP2 | na | 22714 | -0.051 | 0.0644 | No |
| 248 | CTNNA2 | na | 22741 | -0.051 | 0.0648 | No |
| 249 | LAMA4 | na | 23170 | -0.057 | 0.0538 | No |
| 250 | PTGS2 | na | 23198 | -0.057 | 0.0544 | No |
| 251 | CCNE1 | na | 23221 | -0.058 | 0.0552 | No |
| 252 | ARNT | na | 23256 | -0.058 | 0.0556 | No |
| 253 | CASP8 | na | 23310 | -0.059 | 0.0555 | No |
| 254 | IKBKB | na | 23356 | -0.060 | 0.0556 | No |
| 255 | STAT1 | na | 23384 | -0.060 | 0.0563 | No |
| 256 | PTEN | na | 23399 | -0.060 | 0.0574 | No |
| 257 | VHL | na | 23429 | -0.061 | 0.0580 | No |
| 258 | LAMA2 | na | 24087 | -0.071 | 0.0406 | No |
| 259 | ERBB2 | na | 24092 | -0.071 | 0.0422 | No |
| 260 | CTBP1 | na | 24172 | -0.072 | 0.0417 | No |
| 261 | PTCH2 | na | 24205 | -0.072 | 0.0425 | No |
| 262 | SKP2 | na | 24233 | -0.073 | 0.0435 | No |
| 263 | MDM2 | na | 24258 | -0.073 | 0.0445 | No |
| 264 | KLK3 | na | 24301 | -0.074 | 0.0451 | No |
| 265 | CDKN2B | na | 24464 | -0.077 | 0.0423 | No |
| 266 | PLCG2 | na | 24577 | -0.078 | 0.0409 | No |
| 267 | CBL | na | 24630 | -0.079 | 0.0413 | No |
| 268 | WNT5A | na | 24650 | -0.080 | 0.0427 | No |
| 269 | FGFR3 | na | 24695 | -0.080 | 0.0434 | No |
| 270 | CEBPA | na | 24708 | -0.081 | 0.0450 | No |
| 271 | MAPK9 | na | 24826 | -0.082 | 0.0436 | No |
| 272 | CASP3 | na | 24828 | -0.083 | 0.0456 | No |
| 273 | CREBBP | na | 24831 | -0.083 | 0.0475 | No |
| 274 | TGFB2 | na | 24944 | -0.084 | 0.0463 | No |
| 275 | AR | na | 25031 | -0.086 | 0.0459 | No |
| 276 | BRAF | na | 25048 | -0.086 | 0.0475 | No |
| 277 | KRAS | na | 25083 | -0.087 | 0.0486 | No |
| 278 | LAMA5 | na | 25084 | -0.087 | 0.0507 | No |
| 279 | SMAD2 | na | 25145 | -0.088 | 0.0511 | No |
| 280 | MTOR | na | 25211 | -0.089 | 0.0514 | No |
| 281 | PLCG1 | na | 25238 | -0.090 | 0.0528 | No |
| 282 | FGF8 | na | 25299 | -0.091 | 0.0532 | No |
| 283 | TRAF4 | na | 25334 | -0.091 | 0.0544 | No |
| 284 | NTRK1 | na | 25343 | -0.091 | 0.0564 | No |
| 285 | PIK3CG | na | 26210 | -0.109 | 0.0339 | No |
| 286 | RALGDS | na | 26371 | -0.112 | 0.0320 | No |
| 287 | COL4A1 | na | 26628 | -0.117 | 0.0274 | No |
| 288 | PTCH1 | na | 26724 | -0.119 | 0.0275 | No |
| 289 | SPI1 | na | 26751 | -0.120 | 0.0296 | No |
| 290 | SOS1 | na | 26852 | -0.122 | 0.0297 | No |
| 291 | AKT2 | na | 27111 | -0.128 | 0.0253 | No |
| 292 | WNT16 | na | 27162 | -0.130 | 0.0270 | No |
| 293 | WNT10B | na | 27303 | -0.133 | 0.0261 | No |
| 294 | TRAF3 | na | 27488 | -0.138 | 0.0241 | No |
| 295 | IGF1R | na | 27632 | -0.142 | 0.0234 | No |
| 296 | PIK3R1 | na | 27720 | -0.144 | 0.0244 | No |
| 297 | FGF3 | na | 27771 | -0.145 | 0.0264 | No |
| 298 | FOS | na | 27908 | -0.149 | 0.0261 | No |
| 299 | MMP2 | na | 28143 | -0.155 | 0.0230 | No |
| 300 | CCNE2 | na | 28205 | -0.157 | 0.0250 | No |
| 301 | DAPK1 | na | 28371 | -0.161 | 0.0242 | No |
| 302 | MECOM | na | 28559 | -0.166 | 0.0227 | No |
| 303 | PRKCG | na | 28978 | -0.178 | 0.0149 | No |
| 304 | FASLG | na | 29443 | -0.193 | 0.0061 | No |
| 305 | RET | na | 29684 | -0.200 | 0.0040 | No |
| 306 | E2F2 | na | 30000 | -0.211 | -0.0001 | No |
| 307 | FGF9 | na | 30736 | -0.238 | -0.0157 | No |
| 308 | WNT2B | na | 30926 | -0.244 | -0.0153 | No |
| 309 | CBLB | na | 31517 | -0.270 | -0.0259 | No |
| 310 | PAX8 | na | 31897 | -0.288 | -0.0299 | No |
| 311 | FLT3 | na | 31973 | -0.292 | -0.0250 | No |
| 312 | FGF18 | na | 32061 | -0.297 | -0.0203 | No |
| 313 | RARB | na | 32373 | -0.318 | -0.0217 | No |
| 314 | RAC3 | na | 32406 | -0.320 | -0.0148 | No |
| 315 | FGF17 | na | 32598 | -0.334 | -0.0123 | No |
| 316 | LAMB4 | na | 32718 | -0.344 | -0.0074 | No |
| 317 | FGF5 | na | 32744 | -0.346 | 0.0003 | No |
| 318 | PDGFRB | na | 32781 | -0.349 | 0.0077 | No |
| 319 | IGF1 | na | 33039 | -0.370 | 0.0092 | No |
| 320 | PDGFRA | na | 33905 | -0.477 | -0.0044 | No |
| 321 | FN1 | na | 34246 | -0.555 | -0.0008 | No |
| 322 | PIK3R5 | na | 34383 | -0.611 | 0.0100 | No |
Table: GSEA details [plain text format]

  

Fig 2: KEGG\_PATHWAYS\_IN\_CANCER      
 Blue-Pink O' Gram in the Space of the Analyzed GeneSet

  

Fig 3: KEGG\_PATHWAYS\_IN\_CANCER: Random ES distribution      
 Gene set null distribution of ES for **KEGG\_PATHWAYS\_IN\_CANCER**
